# Validation of the conceptual research utilization scale: an application of the standards for educational and psychological testing in healthcare

**DOI:** 10.1186/1472-6963-11-107

**Published:** 2011-05-19

**Authors:** Janet E Squires, Carole A Estabrooks, Christine V Newburn-Cook, Mark Gierl

**Affiliations:** 1 Clinical Epidemiology Program, Ottawa Hospital Research Institute, Ottawa, Ontario, Canada; 2 Faculty of Nursing, University of Alberta, Edmonton, Alberta, Canada; 3 Department of Educational Psychology, University of Alberta, Edmonton, Alberta, Canada

## Abstract

**Background:**

There is a lack of acceptable, reliable, and valid survey instruments to measure conceptual research utilization (CRU). In this study, we investigated the psychometric properties of a newly developed scale (the CRU Scale).

**Methods:**

We used the *Standards for Educational and Psychological Testing *as a validation framework to assess four sources of validity evidence: content, response processes, internal structure, and relations to other variables. A panel of nine international research utilization experts performed a formal content validity assessment. To determine response process validity, we conducted a series of one-on-one scale administration sessions with 10 healthcare aides. Internal structure and relations to other variables validity was examined using CRU Scale response data from a sample of 707 healthcare aides working in 30 urban Canadian nursing homes. Principal components analysis and confirmatory factor analyses were conducted to determine internal structure. Relations to other variables were examined using: (1) bivariate correlations; (2) change in mean values of CRU with increasing levels of other kinds of research utilization; and (3) multivariate linear regression.

**Results:**

Content validity index scores for the five items ranged from 0.55 to 1.00. The principal components analysis predicted a 5-item 1-factor model. This was inconsistent with the findings from the confirmatory factor analysis, which showed best fit for a 4-item 1-factor model. Bivariate associations between CRU and other kinds of research utilization were statistically significant (p < 0.01) for the latent CRU scale score and all five CRU items. The CRU scale score was also shown to be significant predictor of overall research utilization in multivariate linear regression.

**Conclusions:**

The CRU scale showed acceptable initial psychometric properties with respect to responses from healthcare aides in nursing homes. Based on our validity, reliability, and acceptability analyses, we recommend using a reduced (four-item) version of the CRU scale to yield sound assessments of CRU by healthcare aides. Refinement to the wording of one item is also needed. Planned future research will include: latent scale scoring, identification of variables that predict and are outcomes to conceptual research use, and longitudinal work to determine CRU Scale sensitivity to change.

## Background

Research utilization refers to the "process by which specific research-based knowledge (science) is implemented in practice" [1]. In recent years, we have gained insights into the construct of research utilization, in particular as it applies to nursing practice [2,3]. Despite these gains, little has been done to develop robust (reliable and valid) measures of research utilization in nursing and in healthcare generally. In fact, access to such measures is a persistent and unresolved problem in the research utilization field [1,4,5]. Obtaining reliable and valid assessments of research utilization in healthcare settings is essential for several reasons. First, they are necessary to empirically verify the assumption that patient outcomes are sensitive to varying levels of research utilization by healthcare providers. Secondly, and importantly, robust measurement of research utilization is needed to better understand the latent construct, including its causal predictors and effects. These causal mechanisms will inform the development and evaluation of interventions to improve patient care by increasing healthcare providers' use of research findings in clinical practice.

Research utilization is a multidimensional construct that consists of three kinds of research use: instrumental, conceptual, and symbolic (or persuasive) [2,6], each of which, is believed to represent a single concept. *Instrumental research utilization *is a direct use of research knowledge. It refers to the concrete application of research in clinical practice, either in making specific decisions or as knowledge to guide specific interventions related to patient care. For instrumental use, the research is often translated into a material and useable form (e.g., a policy, protocol or guideline) [2,6]. C*onceptual research utilization *(CRU) refers to the cognitive use of research where the research findings may change one's opinion or mind set about a specific practice area but not necessarily one's particular action. It is an indirect application of research knowledge [2,6]. An example of CRU would be the use of knowledge on the importance of Family-Centered Care to guide clinical practice. *Symbolic (or persuasive) research utilization *is the use of research knowledge as a political tool in order to influence policies and decisions *or *to legitimate a position [2,6]. For instance, using a research-based pain assessment to advocate for appropriate medication orders would be an example of symbolic research utilization. Estabrooks [2] embarked on a study to explore and provide some empirical support for this conceptual structure of research utilization and concluded that "instrumental, conceptual, and persuasive research utilization exist and that a global measure of research utilization (overall research utilization) may be defensible" (p. 203). Estabrooks [2] defined *overall research utilization *as the use of any kind of research in any way in clinical practice and conceptualized it as an omnibus and multidimensional construct [2,7].

### Conceptual Research Utilization

While the number of studies examining research utilization has increased significantly in the past decade, the majority continue to examine research utilization as a general construct or instrumentally [8]. Conceptual use of research findings has received little attention. The concept of conceptual research utilization (CRU) originated in the 1970's in investigations of how social science policy makers 'use research'. It was discovered that policy makers most frequently use research, not to act upon a situation, but rather to *inform *their decision-making process [9-12]. As a result, the concept of CRU is believed to be more reflective of the process of research utilization at the individual practitioner level than are the other (i.e., instrumental, symbolic) kinds of research utilization [12-14]. Furthermore, in studies where multiple kinds of research utilization have been assessed, regardless of the study's context, CRU often occurred more frequently then did the other kinds of research utilization or overall measures of research utilization [7,15-19].

We located 11 articles published between 1989 and 2009, whose authors had measured CRU by nursing care providers. All studies measured CRU by registered nurses and/or licensed practical nurses [2,7,15-24], while one study also measured CRU by healthcare aides (unregulated nursing service delivery providers) [19]. The most frequently used measure of CRU (used in 7 of the 11 articles) was a single item developed by Estabrooks [2] to measure CRU by registered nurses. The measure consists of a definition of CRU, examples of CRU, which are then followed by a single item that asks respondents to indicate, on a 7-point frequency scale (1 'never' to 7 'nearly every shift'), how often they used research in the way described in the definition and examples in the past year. One study [21] used the same question but with a 5-point frequency scale (1 'never' to 5 'very often'). The findings from these studies have shown individual variability in the reported CRU score as well as consistency across the various studies (when the question is asked of professional nurses). Connor [19] also reported variability in the reported CRU score when this item is used with healthcare aides. However, a recent study conducted in two long-term care facilities in Western Canada to pilot test a survey designed to measure organizational context and research utilization revealed this same CRU item lacked validity when administered to healthcare aides. In this study, the healthcare aides expressed difficulty comprehending the meaning of CRU [25] as expressed in the definition and examples. As a result, we developed a new multi-item scale - the *Conceptual Research Utilization Scale *(hereafter called the CRU scale) -to measure conceptual research use by healthcare aides.

### Psychometric Testing

Assessment of the psychometric properties of a new instrument involves testing the instrument for: (1) validity, (2) reliability, and (3) acceptability [26-28]. *Validity *refers to the extent to which a measure achieves the purpose for which it is intended, and is determined by the "degree to which evidence and theory support the interpretations of test scores entailed by proposed users of tests" [29] (p. 9). *Reliability *refers to the consistency of measurement obtained when using an instrument repeatedly on a population of individuals or groups [29]. *Acceptability *refers to ease of use of an instrument [27]. In this study, we assessed the validity, reliability, and acceptability of scores obtained on the CRU scale when completed by healthcare aides in residential long-term care settings (nursing homes). We used the *Standards for Educational and Psychological Testing *(the *Standards*) [29] to guide our validity assessment.

### The *Standards*

The *Standards*, considered best practice in the field of psychometrics [30], follow closely the work of American psychologist Samuel Messick [31-33], who viewed validity as a unitary concept with all validity evidence contributing to construct validity. Validation, in this framework, involves accumulating evidence from four sources: (1) content; (2) response processes; (3) internal structure; and (4) relations to other variables. The source(s) of evidence sought for any particular validation is determined by the desired interpretation(s) [34,35]. Since this is the first validation study on the CRU scale and thus largely exploratory in nature, we sought evidence from all four sources at both the scale and item level. Item level analysis was conducted to provide insight into any revisions to the scale that may be needed. Content evidence is usually the first type of evidence sought in the assessment of a new instrument. In this study, however, it comprised the second type of validity evidence; we sought and used response processes evidence to modify the scale before performing a formal content validity assessment and pilot testing the scale, and therefore discuss it (responses processes evidence) first.

***Response processes evidence ***refers to empirical evidence of the fit between the concept under study (CRU) and the responses given by respondents on the item(s) developed to measure the concept [29]. Response processes evidence can come in a variety of forms but is most often derived from observations or interviews employed to determine if an individual's behavior or verbal explanation(s) are congruent with their responses to an instrument item/question [36]. ***Content evidence ***refers to the extent to which the items included in an instrument adequately represent the content domain of the concept of interest [27]. Content evidence is largely a matter of judgment, and can involve: (1) *a priori *efforts by scale developers (i.e., careful conceptualization through development or selection of items that are based on existing literature or theory) and, (2) *a posteriori *efforts (after the scale is developed) using a panel of content experts to evaluate the relevance of the scale's items to the concept of interest [37,38]. ***Internal structure evidence ***refers to the relationships between the items in an instrument. Factor analytic approaches are frequently used to assess internal structure. Finally, ***relations to other variables evidence***, refers to analyses of the relationships between scores obtained for the concept of interest (CRU) and variables external to the concept. External variables may include measures, which the concept is expected to predict, as well as other scales hypothesized to measure the same concept, and related or different concepts. No one kind of *relations to other variables *evidence is always desired; the specific evidence sought will depend on the score interpretations desired. This type of evidence is most often expressed in the form of bivariate correlations, statistical (predictive) models, and/or multi-group-comparisons [29].With respect to the CRU scale, there is a paucity of empirical studies examining the relations between CRU and external variables, in turn restricting the amount of *relations to other variables *evidence that could be obtained in this study. However, evidence does exist to suggest that CRU (when assessed with professional nurses) is: (1) correlated with instrumental research utilization and symbolic research utilization [2]; and, (2) a cause of research utilization (indicated by 'overall research utilization') [2,7]. Confirmation of these associations, using scores obtained with the new CRU scale, will provide beginning *relations to other variables *evidence.

## Methods

### CRU Scale Development

The CRU scale was developed as part of a larger research program - the *Translating Research In Elder Care *(TREC) program [39]. Development of the CRU scale was guided by two key principles: (1) brevity - the scale was required to be less than 10 items so that it could be easily administered as part of a larger survey in busy resource-stretched nursing homes; and, (2) generality - the scale was intended to capture the concept of CRU broadly so that it could be administered in a wide range of nursing home settings. Therefore, terminology that is specialty (e.g., dementia care) and culture (e.g., Canadian or American) specific was intentionally avoided. The scale items were derived from an 18-item checklist designed by Stetler and Caramanica [23] to evaluate an evidence-based practice initiative. Items were selected that correspond to how CRU is defined, that is, the cognitive use of research where the research findings may change one's opinion or mind set about a specific practice area but not necessarily one's particular action [2,6]. Six items (later reduced to five items) from the Stetler and Caramanica [23] checklist were selected and modified (with permission from the checklist developers) for use with nursing care providers in nursing homes. The items were selected to be reflective indicators of CRU yet explicitly non-redundant items. The scale underwent several feasibility iterations with healthcare aides in two nursing homes in Alberta, Canada before being tested more fully in the TREC study. The final version of the scale, presented in Additional File 1, contained five items and asked respondents to score how often best practice knowledge led to the activities reflected in each of the items. 'Best practice' was used for 'research' in the scale as this reflects terminology commonly used by healthcare aides. A 5-point Likert-type frequency scale was used where 1 indicated 'never', 2 indicated 'rarely', 3 indicated 'occasionally', 4 indicated 'frequently' and 5 indicated 'very frequently'. Higher scores indicated a higher level of CRU.

### Sampling, Data Collection, and Analysis

We used three samples to conduct the validation study presented in this paper. A description of the samples, data collection and analytic approaches taken are described next.

### Sample 1

#### Description and Data Collection

The first sample collected *response processes *validity evidence from healthcare aides to determine fit between the items comprising the scale and the concept of CRU. Participants consisted of 10 healthcare aides from two general units in two nursing homes in Alberta Canada. All healthcare aides employed on the two units were invited to participate. The healthcare aides completed the CRU scale during work time in a private room (located outside of their work unit). Data collection occurred in three iterations (n = 1, n = 2, and n = 7 healthcare aides participated in each iteration respectively) between December 6, 2008 and December 21, 2008. The data collection process included reviewing a study information letter with each healthcare aide; obtaining signed informed consent; administration of the CRU scale by a member of research team by reading it aloud to the healthcare aide; and an informal conversation with a research team member following completion of the scale. All questions or comments regarding the scale made by the healthcare aides were recorded.

#### Data Analysis

Following each iteration, two research team members analyzed all comments recorded during the scale administration and informal conversation sessions using content analysis. Findings from the content analysis were then discussed and used to inform any changes to the scale items/response options prior to the next testing (iteration). The final form of the CRU scale (post-iteration 3), presented in Additional File 1, was subject to further validity assessments of: content (sample 2, expert panel assessment) and internal structure and relations to other variables (sample 3, pilot test).

### Sample 2

#### Description and Data Collection

The second sample was comprised of an international panel of experts in research utilization in nursing, and was used to collect *content *validity evidence. This phase of the study occurred concurrently with the pilot test (sample 3). A content validity survey was developed, which asked respondents (experts) to rate each of the five items comprising the CRU scale with respect to their relevance to the concept of CRU. A 4-point Likert scale was provided: 1 'not relevant'; 2 'item needs some revision'; 3 'relevant but needs minor revision'; and 4 'very relevant'. This is a modified version of Davis' scale [40], which has been used in past studies examining item to concept relevance (content validity) [27]. Respondents were also given the option of providing comments in an open-ended field on the survey. The survey was sent electronically to 11 international experts in the research utilization field, identified through our knowledge of the field and a literature search. A minimum of five experts are recommended for an expert panel content validity assessment [41].

#### Data Analysis

There are numerous methods of quantifying agreement on *content *relevance. We chose to use content validity index (CVI) scores and intraclass correlation (ICC). CVI scores allow for item-level assessments (in addition to scale level assessments) and are more easily interpreted and understood than are other methods of agreement [37]. For this reason, CVI was our primary method for quantifying agreement from the expert panel. First, for each item in the CRU scale we calculated CVI scores (referred to as I-CVI). The I-CVI was calculated as follows: the number of experts giving a rating of either 3 or 4 (relevant) divided by the total number of experts scoring the item [37]. The accepted standard in the literature for an I-CVI is 0.78 [37,42]. Second, for the full CRU scale (all five items together) we calculated a CVI score (referred to as S-CVI). The S-CVI was calculated using two methods: (1) universal agreement (referred to as S-CVI/UA); and, (2) average or mean expert proportion (referred to as S-CVI/avg). The S-CVI/UA was calculated as the number of items that the experts gave a rating of either 3 or 4 (relevant) divided by the total number of item ratings provided by the experts [37]. An S-CVI rating of 0.80 is considered acceptable [37,40]. Because the S-CVI/UA tends to decrease when greater than 2 experts are used, we also calculated the mean expert proportion (S-CVI/avg) as recommended by Polit and Beck [37]. The mean expert proportion refers to the average proportion of items rated as relevant across the experts, and was calculated by taking the mean of the proportion of items that were rated either 3 or 4 (relevant) across the nine experts. A value of .80 or higher is considered acceptable [37].

We also calculated the ICC (2,1). Intraclass correlations describe how strongly units in the same group resemble each other and are often reported as an assessment of consistency of quantitative measurements made by different observers observing the same behavior or measuring the same quantity. With respect to instrument content validity, this refers to ratings made by a number of experts on the relevance of an item to a concept (CRU).

### Sample 3

#### Description and Data Collection

The third sample was used to collect evidence on: (1) *validity - internal structure evidence*; (2) *validity - relations to other variables evidence*; (3) *reliability*; and, (4) *acceptability*. For this phase, a sub-analysis of data collected for the TREC program was used. TREC is a multi-level (provinces, regions, facilities, units within facilities, individuals) and longitudinal research program designed to examine the impact of organizational context on research utilization by healthcare providers and the subsequent impact of research utilization on outcomes (e.g., resident and staff health) in nursing homes across the Canadian Prairie Provinces. Data used in this paper come from the TREC survey, in which the CRU scale is embedded. Data were obtained from healthcare aides employed in 30 urban nursing homes that completed the TREC survey during the project's first year of data collection (July 2008 - June 2009). The 30 nursing homes were selected using stratified random sampling (i.e., stratified by healthcare region, owner operational model, and size). Healthcare aides within each nursing home were recruited using a volunteer, census-like sampling technique. Inclusion criteria included: (1) ability to identify a unit where they have worked for at least 3 months; and, continue to work, and (2) work a minimum of 6 shifts per month on this unit. Additional details on the sampling employed in the original (TREC) study can be found elsewhere [43].

We assessed for significant associations between the scores obtained on the CRU scale and each of the CRU items with respect to healthcare aide selected demographic variables (age and first language) to determine homogeneity of the sample prior to conducting our psychometric assessment. No significant differences were found by age (*p *> 0.05). Healthcare aides with English as their first language however scored significantly lower on all five CRU scale items in comparison to healthcare aides whose first language was not English (independent sample t-test, *p *< 0.05) (See Additional File 2). Because we desired a homogenous sample to conduct the initial psychometric analysis of the scale, we chose to conduct the analyses on healthcare aides with English as their first language (n = 707 cases, n = 697 cases using listwise deletion). A summary of the demographic characteristics of sample 3 is presented in Table 1.

**Table 1:** Sample 3 Characteristics (n = 707)

Demographic Characteristic		n (%)
	Male	34 (4.8%)
	
Gender	Female	668 (94.5%)
	
	*Missing Values*	5 (0.7%)

	<20 years	11 (1.6%)
	
	20-29 years	108 (15.3%)
	
	30-39 years	126 (17.8%)
	
Age	40-49 years	212 (30.0%)
	
	50-59 years	184 (26.0%)
	
	60-69 years	65 (9.2%)
	
	>70 years	0 (0%)
	
	*Missing Values*	1 (0.1%)

Education Level	High School *Missing Values*	615 (87.0%) 2 (0.3%)
	
	HCA Certificate *Missing Values*	592 (83.7%) 0

Shift Worked Most of the Time	Day Shift	373 (52.8%)
	
	Evening Shift	226 (32.0%)
	
	Night Shift	108 (15.3%)
	
	*Missing Values*	0

English as a First Language	Yes	707 (51.7%)

	No	659 (48.2%)

	*Missing Values*	1 (0.1%)

		**Mean (SD)**

Number of Years Worked as a Healthcare Aide		11.8 (9.65)

Number of Years Worked on Unit		4.8 (5.58)

Hours Typically Worked in Two Weeks		65.30 (18.09)

#### Data Analysis

Since this was the first field assessment of the CRU Scale, our assessment was largely exploratory in nature. Therefore, to examine the underlying dimensional structure of the CRU Scale, we performed: (1) item-total statistics (using PASW Version 18.0 [44]), (2) principal component analysis (PCA) (using PASW Version 18.0 [44]), and (3) confirmatory factor analysis (CFA) (using LISREL [45]). Missing values, which were limited, were treated as such with no substitution or imputation of estimated values. From the item-total statistics, items were considered for removal and/or revision if any of the following three criteria were met: (1) the item correlated with the total CRU scale score below 0.30 (using corrected item-total correlations); (2) the item caused a substantial drop (10% or more) in the scale Cronbach's alpha score when removed; and, (3) the items were highly correlated with each other (*r *> .80) [26,46]. The scree plot and Kaiser-criterion (eigenvalue >1) were considered in determining the optimal number of factors from the PCA [47,48].

The items comprising the CRU Scale were selected during scale development to be similar yet explicitly non-redundant items, and hence the factor-structured models traditionally employed to assess internal structure are not precisely correct, though the similarity of items within the CRU scale renders the factor structure the most appropriate of the available model structures. We ran three confirmatory factor models. Model 1 was comprised of the five items loading onto one factor (CRU). When Model 1 failed to support a strict unidimensional structure, we did a more detailed investigation by setting up two alternate models: Model 2 comprised the five items loading onto one factor (CRU) but with correlated measurement errors between two sets of items based on error theory, and Model 3 was a modified version of Model 2, whereby one item was dropped from the model (based on theory and statistical measures). We assessed model-data fit of all three models using the chi-square statistic and three fit indices: (1) the root mean square of approximation (RMSEA); (2) the standardized root mean square residual (SRMSR); and, (3) the comparative fit index (CFI). The chi-square statistic tests whether a model-implied covariance matrix is consistent with a sample covariance matrix; a non-significant chi-square value implies acceptable fit. A RMSEA < 0.06 and SRMSR < 0.09 [28,49] and a CFI value > 0.90 [28,50] indicate 'close fit'.

To examine *relations to other variables *validity we conducted the following analyses: (1) bivariate correlations between each CRU scale item and instrumental, persuasive, and overall research utilization; (2) assessment for change in mean scores for each CRU item at increasing levels of instrumental, persuasive, and overall research utilization; and, (3) a multivariate linear regression model with overall research utilization was the dependent variable.

To assess the reliability of the CRU scale we calculated three internal consistency coefficients: (1) Cronbach's alpha; (2) Guttman split-half reliability; and, (3) Spearman-Brown reliability. Coefficients can range from 0 to 1; a coefficient of 0.70 is considered acceptable for newly developed scales while 0.80 or higher is preferred and indicates the items may be used interchangeably [26,27]. We assessed acceptability of the CRU scale by evaluating: (1) missing-value rates; and, (2) the average length of time it took for the healthcare aides to complete the scale [26-28].

### Ethics

Ethics approval was obtained from the Human Research Ethics Board at the University of Alberta (Canada). Operational and administrative approvals were obtained from the research facilitation committee overseeing the participating facilities and the TREC research program.

## Results

### Validity Assessment

#### Response Process Evidence

Revisions were made to several of the items as a result of this phase of the study. *First*, general wording changes were made to make the items more reflective of nursing homes and the work of healthcare aides. Examples of wording changes included using the word 'resident' instead of 'patient'. General wording changes were also made to the stem (lead-in) for the 5 items. For example, we changed the word 'research' to 'best practice' to reflect terminology commonly used and understood by healthcare aides. *Second*, item 3 was reworded from 'help to change your attitudes or beliefs about how to care for residents' to 'help to change your mind about how to care for residents' to increase clarity. *Third*, one of the original six items was removed. The item 'help you plan your workday better' was removed because its interpretation by the healthcare aides (according to the comments they provided) was not congruent with the concept of CRU. *Fourth*, changes were made to the response options used. We began with a 5-point frequency scale (1 '10% or less of the time' to 5 'almost 100% of the time'). However, the healthcare aides found these options difficult to interpret. In iteration 2 we trialed a 5-point Likert scale (1 'never' to 5 'almost always'), which the healthcare aides interpreted more easily. Discussions with healthcare aides following iteration 2 resulted in one final change - response option 5 was changed from 'almost always' to 'very frequently'. The revised CRU scale (stem, items, and response options) was then tested in iteration 3; no additional changes were required, providing evidence of fit between the construct of CRU and the five items as they were interpreted by healthcare aides (i.e., response processes validity evidence).

#### Content Evidence

A total of 10 (of 11) content validity surveys were returned for a response rate of 91%. One returned survey was not usable due to missing data, leaving an analytic sample of n = 9. The nine experts represented five countries: Canada (n = 3), United Kingdom (n = 2), Sweden (n = 2), United States (n = 1), and Australia (n = 1). Table 2 summarizes the content validity index (CVI) scores calculated from the responses provided to the content validity survey. Items 2 through 5 displayed acceptable (>0.78) I-CVI scores while item 1 (give new knowledge or information) was below the accepted standard with a score of 0.55. Several members of the expert panel also provided additional comments on item 1. One expert stated that there was some "uncertainty" around item 1. Another expert stated there was "conceptual overlap" between items 1 and 4 (item 4 - give you new ideas). Two experts also suggested that item 1 could reflect both instrumental and conceptual research utilization.

**Table 2:** Content Validity Index (for relevance)

Item	Expert									Number in agreement	Item CVI
	**1**	**2**	**3**	**4**	**5**	**6**	**7**	**8**	**9**		

**Item #1**: Give new knowledge or information	3	4	3	2	1	1	4	3	2	5	0.55

**Item #2**: Raise awareness	4	4	4	4	3	4	4	4	2	8	0.89

**Item #3**: Help change your mind	4	4	3	4	4	4	4	4	4	9	1

**Item #4**: Give new ideas	4	3	3	4	4	4	4	4	4	9	1

**Item #5**: Help make sense of things	4	4	4	4	4	4	4	3	2	8	0.89

Proportion Relevant	1.00	1.00	1.00	.80	.80	.80	1.00	1.00	.40		

Mean I-CVI = .844

Mean I-CVI (item 1 removed) = .920

S-CVI/UA = .40

S-CVI/UA = (item 1 removed) = .40

S-CVI/avg = .87

S-CVI/avg (item 1 removed) = .94

The scale content validity/universal agreement (S-CVI/UA) score was 0.40, indicating low universal agreement on the scale by *all *experts (Table 2). The alternative measure, the S-CVI/avg (i.e., average proportion relevant) and was 0.87, which exceeded the accepted standard of 0.80 [37]. Given the low relevance score assigned to item 1 and additional comments provided regarding this item, for exploratory purposes, we also calculated the S-CVI with item 1 removed (i.e., on a 4-item scale). The resulting S-CVI/UA was unchanged and S-CVI/avg increased slightly to 0.94. Similar findings were shown when the ICC (2,1) coefficient (a measure of absolute agreement) was calculated for the five-item scale; a value of 0.317 was obtained (0 indicates no agreement and 1 indicates perfect agreement). ICC (2,1) increased substantially when item 1 was removed from the scale (increased to 0.793). Overall, these findings provide support for acceptable content validity of the CRU scale generally (CVI) and items 2 through 5 specifically (CVI and ICC).

#### Internal Structure Evidence

A total of 1367 healthcare aides (representing 73% of those eligible to participate) working in 97 units in the 30 nursing homes completed the TREC survey. The Intraclass correlation 1, ICC(1), estimate for the data indicated that a degree of agreement existed around the group (unit and nursing home) mean for the CRU scale score (ICC1 = .1352 and .1354 when scores are aggregated to unit and nursing home levels respectively). This level of perpetual agreement however is not substantial indicating CRU is largely an individual level variable; best analyzed using classical psychometric approaches.

##### Outliers

Prior to conducting analyses to assess the internal structure of the CRU scale, we examined sample 3 data for univariate and multivariate outliers. To assess for univariate outliers the frequency distributions of each scale item was examined; values greater than 3 standard deviations from the mean indicate univariate outliers [53]. Screening for multivariate outliers was by calculation of the Mahalanobis distance scores for all cases (*D*^2^_*i*_); *D*^2 ^probability < 0.001 indicate multivariate outliers [54]. No outliers were identified, and therefore, all cases were retained for the remaining analyses.

##### Item-Total Statistics

To test for scale homogeneity, corrected item total correlations for the items were calculated. All corrected item-total correlations exceeded the accepted cutoff of 0.30 indicating each item was related to the overall scale [26] (See Table 3). Inter-item correlations (data not shown) were also within acceptable ranges (less then 0.80) for all pairs of items [26]. Therefore, all five items were retained and entered into the PCA and CFA.

**Table 3:** Item Characteristics (n = 697^1^)

Item	Corrected Item- Total Correlations	**Factor Loading**^**2**^
**Item #1**: Give new knowledge or information	0.722	0.688

**Item #2**: Raise awareness	0.782	0.756

**Item #3**: Help change your mind	0.666	0.610

**Item #4**: Give new ideas	0.788	0.759

**Item #5**: Help make sense of things	0.749	0.716

^1^listwise deletion resulted in the removal of 10 cases for a final sample size of 707-10 = 697 cases

^2^Eigenvalue = 3.529; variance explained = 70.579%

##### Principal Components Analysis (PCA)

Before running the PCA, the Kaiser-Meyer-Olkin measure of sampling adequacy and the Bartlett test of sphericity were assessed to determine if the data was appropriate for PCA [55,56]. The large value calculated by the Bartlett's test of sphericity indicated that the correlation matrix for the five items was not an identity matrix (χ^2 ^= 2012.702, *df *= 10, p < 0.001), and the Kaiser-Meyer-Olkin measure indicated acceptable sampling adequacy (0.866). From the PCA, one-dominant factor (eigenvalue = 3.529 accounting for 70.6% of variance and covariance in the items) was extracted from the scale items. Visual inspection of the scree plot (plot of the eigenvalues) was consistent with this finding. Factor loadings were substantial, ranging from 0.610 to 0.759 (Table 3).

##### Confirmatory Factor Analysis (CFA)

Factor loadings for all three CFA models are displayed in Table 4. The one-dominant factor model that emerged from the PCA was somewhat inconsistent with the findings from the CFA. While all parameters (i.e., factor loadings) in the CFA were significant in a positive direction as hypothesized, the χ^2 ^test statistic did not support a strict 1-factor model (χ^2 ^= 69.53, *df *= 5, *p *= 0.0). The RMSEA (0.140) did not support close fit but SRMSR (0.03) and CFI (0.977) did support close fit. Based on these findings, we rejected the simple 1-factor model.

**Table 4:** Confirmatory Factor Analyses (n = 697^1^)

	Factor Loadings (Completely Standardized Solution)		
**Item**	**Model 1**	**Model 2**	**Model 3**

**Item #1**: Give new knowledge or information	0.788	0.741	

**Item #2**: Raise awareness	0.853	0.815	0.815

**Item #3**: Help change your mind	0.708	0.695	0.703

**Item #4**: Give new ideas	0.836	0.844	0.846

**Item #5**: Help make sense of things	0.799	0.822	0.819

^1^listwise deletion resulted in the removal of 10 cases for a final sample size of 707-10 = 697 cases.

Modification indices, which suggest how much the χ^2 ^test is expected to improve if a fixed parameter is freed to be estimated, suggested freeing seven of the possible ten measurement error covariances in the model (the three exceptions were the error covariances for: items 1 and 5; items 2 and 3; and items 4 and 5). A careful re-examination of the five items comprising the scale revealed a level of content overlap with respect to two pairs of items: items 1 (give new knowledge or information) with 2 (raise awareness); and, items 3 (help change your mind) with 4 (give new ideas). We therefore considered the possibility that systematic error variance may be causing these items to group together beyond their dependence on one principal factor. We hypothesized that in addition to the five items loading onto a single factor; there would be error covariances for items 1 and 2, and items 3 and 4. We chose not to allow the errors on the remaining five pairs of items identified in the modification indices to correlate because they did not match this error theory. This error theory was also supported statistically; these two pairs of items displayed the largest standardized residuals and modification indices among all possible pairs of items (see Additional File 3).

Model 2, where we correlated errors on items 1 and 2, and items 3 and 4, resulted in improved and a marginally acceptable fit (χ^2 ^= 6.86, *df *= 3, *p *= 0.075). The close fit statistics also improved (RMSEA = 0.043, SRMSR = 0.009, CFI = 0.999). We concluded based on these findings that the 1-factor model incorporating limited error theory was superior to the strict 1-factor model. However, the need to correlate errors to attain a better-fitting model raised the question of why items that overlap significantly in content are necessary in the scale. As a final modification, we therefore selected to drop item 1 and rerun model 2. We dropped item 1 based on: (1) the error theory (that item 1 had redundancy with item 2), (2) that it (item 1) received the lowest I-CVI score (Table 2), and (3) that it (item 1) displayed a lower factor loading compared to item 2 in the PCA (Table 3) and CFA (Table 4). We tested this model (Model 3 - 1-factor, item 1 removed, correlated error between items 3 and 4). Although it was restricted in testing power with *df *= 1, it resulted in improved fit (χ^2 ^= 2.43, *df *= 1, *p *= 0.119) in comparison to the previous two models. The close fit statistics remained relatively unchanged from model 2 (RMSEA = 0.045, SRMSR = 0.007, CFI = 0.999). A final alternate model would be a three-item scale (without item 1 and one of items 3 or 4). However, such a model would be just identified (*df *= 0) and not testable.

#### Relations to Other Variables Evidence

##### Correlations and Change in Mean Values

The bivariate correlation analysis conducted on the CRU scale items is presented in Table 5. Since this is the first assessment of the CRU scale and largely exploratory in nature, we have elected not to derive a score for a 4-item scale (i.e., a scale without item 1), instead the scale score uses all 5 items. We did this so that we could review all validity evidence on the 5-item scale before deciding on any scale revisions. The CRU items, as well as the total CRU scale score (obtained by taking a mean of the five items), were positively correlated with instrumental research utilization, symbolic research utilization, and overall research utilization (each measured in the TREC survey by single items and scored on a five-point frequency scale from 1 'never' to 5 'almost always'). The magnitude of the associations were low to moderate, and were strongest with symbolic research utilization, followed by overall research utilization and finally instrumental research utilization. The only exception to this trend was with item 3 (help change your mind) where the correlation coefficient was minimally higher with instrumental research utilization compared to overall research utilization.

**Table 5:** Assessment of Relations with Other Variables Validity: Correlation of CRU Items by Increasing Levels of Instrumental, Symbolic, and Overall Research Utilization

CRU Item	Instrumental Research Utilization						Symbolic Research Utilization						Overall Research Utilization					
	
	Pearson *r*	**Level of Research Use**^**1**^					Pearson *r*	**Level of Research Use**^**1**^					Pearson *r*	**Level of Research Use**^**1**^				
						
		1	2	3	4	5		1	2	3	4	5		1	2	3	4	5
**1**	.295**	2.00	2.54	3.33	3.47	3.93	.369**	2.36	3.24	3.39	3.82	4.13	.332**	1.67	2.81	3.05	3.57	3.98

**2**	.263**	2.80	2.85	3.25	3.54	3.93	.361**	2.68	3.27	3.41	3.81	4.17	.279**	2.67	3.06	3.19	3.85	3.97

**3**	.247**	2.40	2.54	2.94	3.21	3.63	.320**	2.36	2.94	3.12	3.47	3.86	.232**	2.33	3.00	2.86	3.28	3.64

**4**	.233**	2.40	3.00	3.17	3.44	3.79	.339**	2.52	3.15	3.36	3.67	4.04	.278**	2.67	2.87	2.99	3.52	3.84

**5**	.191**	3.00	3.31	3.37	3.67	3.93	.318**	2.64	3.39	3.57	3.88	4.15	.317**	3.00	3.19	3.05	3.68	4.07

**Scale Score**	.294**	2.52	2.85	3.21	3.46	3.84	.406**	2.51	3.20	3.37	3.73	4.07	.342**	2.47	2.99	3.02	3.53	3.90

^1 ^= 1'never'; 2'rarely'; 3 'occasionally'; 4 'frequently'; 5 'almost always'

** *p *< 0.01

We also hypothesized that each of the CRU items and the total scale score would show a trend of increasing mean values from lowest to highest levels of the other kinds of research utilization and overall research utilization (Table 5). This trend was largely evident, supporting our hypothesis that as healthcare aides increased their reported use of CRU, they simultaneously increased their reported use of the other kinds of research utilization. Also implicit in this analysis is that while all five CRU items generally conform to this trend, some items (e.g., item 1) have consistently lower starting mean values while other items (e.g., item 5) have higher starting mean values regardless of the kind of research utilization they are being examined against. In addition, some items (e.g., item 2) showed more rapid increases in mean values compared to other items (e.g., item 3).

##### Regression Analysis

Overall research utilization was the dependent variable in the regression analysis; the CRU scale score was entered as an independent variable. A selection of other variables, suggested in past research to be significantly related to and/or predictive of overall research utilization by registered nurses, were also entered as control variables. These variables included: frequency of in-service attendance [7,18]; belief suspension (i.e., the degree to which an individual is able to suspend previously held beliefs in order to implement a research-based change) [7,17,18]; attitude towards research [7,17-19]; instrumental research utilization [2,7]; and, symbolic research utilization [2,7]. The CRU scale score remained a significant predictor of overall research utilization (after controlling for the effects of the other entered covariates) as hypothesized, providing *relations to other variables *validity evidence (Table 6).

**Table 6:** Regression Analysis (Dependent Variable: Overall Research Utilization)

**Model Adjusted *R***^**2**^	Independent Variables	Unstandardized Beta	Standardized Beta	*P*-value
0 .345	**Conceptual Research Utilization (scale score)**	**.109**	**.122**	**.001**
	
	**Instrumental Research Utilization**	**.362**	**.356**	**<.001**
	
	**Symbolic Research Utilization**	**.165**	**.222**	**<.001**
	
	**Belief Suspension**	**.103**	**.105**	**.002**
	
	Attitude towards Research	.101	.060	.060
	
	In-services	.007	.011	.738
	
	**Symbolic Research Utilization**	.165	.221	**<.001**
	
	**Belief suspension**	**.100**	**.101**	**.002**
	
	Attitude towards research	.094	.056	.081
	
	In-services	.006	.010	.757

### Reliability Assessment

Cronbach's alpha for the 5-item CRU scale exceeded the accepted standard (>0.70) for scales intended to compare groups (alpha = 0.894) [26]. By odd-even split of the five items, the Guttman split-half reliability was estimated to be 0.858, and the unequal length Spearman-Brown reliability was 0.894, also exceeding accepted standards [26].

### Acceptability Assessment

The percentage of healthcare aides providing complete data on the CRU scale (i.e., with no missing data) was high at 98.6% (n = 697 of 707 healthcare aides). The average time for completion of the five items was minimal (1 minute and 6 seconds).

## Discussion

### English as First Language

The aim of this paper was to report the psychometric properties of responses obtained with the CRU scale when used with healthcare aides in nursing homes. In line with previous studies [57,58], a substantial number (48%) of the healthcare aides in the TREC study (which comprised our sample 3) were not from Canada and, did not speak English as their first language. This is challenging from a psychometric perspective because a homogenous sample is preferred for psychometric assessments such as factor analysis. There is some evidence to suggest that healthcare aides differ on several psychological concepts, for example, job satisfaction and burnout [58,59], by ethnicity [60] of which first language spoken is a component. In our analysis, we found that healthcare aides who spoke English as their first language reported significantly lower scores on the CRU scale in comparison to healthcare aides who did not report English was their first language. These differences may reflect difficulty generally in understanding of the English language. It may also reflect difficulty in comprehending the concept of CRU and what the items comprising the scale were asking. Another possible explanation for the difference noted in the scores is a social desirability bias effect on part of healthcare aides who do not speak English as their first language since their scores on all items were consistently 'higher' than the scores of aides who did speak English as their first language. The differences in scores may, however, also be a valid discovery that can be explained by examining the specific cultural practices of the healthcare aides that did not speak English as their first language; the vast majority came from a variety of non-western cultures. This could be a fruitful area for future investigation. Although the finding that healthcare aides who speak English as their first language responded differently on the CRU scale compared to healthcare aides who do not speak English as their first language is not fully understood at this time, this study underscores the importance of collecting demographic data on healthcare aides' native language and ethnicity, as well as assessing differences by both variables prior to conducting psychometric analyses. In future research we will conduct additional qualitative work to explore reasons why healthcare aides who do not speak English as their first language score higher on the CRU scale then those that do speak English as their first language. We will also conduct a differential item analysis using item response theory to determine whether the items are biased towards healthcare aides who do or do not speak English as their first language. Bias occurs when one group of individuals has a different probability of endorsing a response category to an item, compared to a second group of individuals, after controlling for the value of the latent trait [61].

### Validity

In this study, we aimed to assess the validity of the CRU scale and each of its items when completed by healthcare aides in nursing homes. A sound validity argument integrates various types of evidence to make a determination about the degree to which existing evidence and theory support the intended interpretations of scale scores for specific uses [29]. The *Standards'*, adopted in this study, focuses on content, response processes, internal structure, and relations to other variables evidence to obtain a unitary and comprehensive perspective of validity. In this framework all validity contributes to construct validity and exists as a matter of degree, meaning interpretations from scores are more or less valid given a specific context. The *Standards' *approach therefore provides an alternative to the traditional conceptualization of validity which views validity as: (1) distinct types (e.g., content, criterion, construct), and (2) existing or not.

In this study, we systematically performed several analyses to seek validity evidence (in each of the four domains comprising the *Standards*) with respect to the scores and interpretations obtained from the CRU scale when completed by healthcare aides in nursing homes. While it does do not provide a complete picture of all aspects of validity, it does provide a much needed first look at several critical issues that need to be addressed before more in-depth validity studies can be undertaken with additional samples.

Content validity is an important source of validity evidence; it is essential to identifying the concept being measured and is an early step in establishing construct validity. We explored content validity in a number of ways. First, we attempted to include a representative sample of items by reviewing the existing literature and modifying previously developed statements designed to capture conceptual use of knowledge in acute care hospitals with professional nurses. Second, before conducting a formal content validity assessment with experts, we assessed the appropriateness of the scale with respondents representative of those for whom it was developed (i.e., healthcare aides). This latter activity is formally labeled as 'response processes' validity evidence in the *Standards*. Based on this analysis, several revisions were made to the scale before it was formally assessed for item-concept relevance (i.e., content validity) with an expert panel. This process (integrating content and response process approaches to validation) illustrates the importance of considering multiple evidence sources. A traditional (more compartmentalized) approach to validity assessment would have resulted in the original items being assessed for relevance by an expert panel without knowledge of misfit between the items (as interpreted by the healthcare aides) and the concept of CRU. However, by adopting the *Standards *approach and letting multiple evidence sources inform one another, we were able to pilot test a form of the CRU scale that produced more valid score interpretations, then would have been used, if a traditional approach to validity assessment was undertaken.

Our validity assessment revealed problems with two of the five items in the CRU Scale: item 1 (give new knowledge or information) and item 3 (help change your mind). The formal (expert) content validity assessment resulted in item 1 (give new knowledge or information) being rated at an unacceptable level overall with respect to its relevance to CRU. Some experts also identified item 1 as having content overlap with the concept of instrumental research utilization. The ICC (2,1) measure of agreement further supported item 1 needing removal and/or revision; ICC (2,1) increased substantially when item 1 was removed from the scale (0.317 with item 1 to 0.793 without item 1). While the bivariate correlation between item 1 and instrumental research utilization was low - moderate (0.295), of the five scale items, it correlated the strongest with instrumental research utilization, lending some empirical support to the expert panel's assessment of the item (that it had content overlap with instrumental research utilization). Other issues with item 1 also emerged in our analysis. For example, item 1 had the second lowest factor loading in the PCA (though still substantial, Table 3), and model fit increased significantly in the CFA when the item was removed from the model. Post-analysis inspection of the item also revealed it to be a 'double-barreled' item, meaning it conveys two ideas: (1) give new knowledge; and, (2) give new information. Such items should be avoided wherever possible in instrument development since endorsement of the item might refer to either or both ideas [62]; however the item was not discovered to be double barreled until after the pilot test. Taken together, these findings suggest removal and/or revision of item 1 is required. Revision of the item so that it represents a single idea may lead to improved fit with the remaining four items. However, it is also possible that item 1 represents a distinguished aspect of CRU (i.e., an aspect not captured by the remaining four items); this would mean CRU is a more complex concept then the literature portrays and is multi-dimensional in nature. If this is confirmed in future research, an additional item group to assess this distinguished aspect of CRU should be developed. Until further research is conducted on item 1 (testing whether rewording the item improves its fit with the remaining four scale items or whether it represents a distinguished aspect of CRU), we recommend only using the four-item version of the scale (*i.e.*, without item 1) in assessments of CRU by healthcare aides.

Item 3 (help change your mind) received a perfect relevance score in the formal content validity assessment (Table 2). However, the healthcare aides experienced difficulty comprehending this item according to our response processes work, which occurred prior to this assessment. Item 3 also exhibited the lowest factor loading of the five items in the PCA and CFA and the lowest corrected item total correlation (Tables 3 and 4). In our assessment of change in mean values with increasing levels of instrumental, persuasive, and overall research utilization, item 3 displayed the least change (Table 5). Combined, these findings indicate the healthcare aides may have had continued difficulty interpreting the item. These findings also demonstrate the importance of taking a comprehensive approach to validity assessment. While the formal content assessment revealed a perfect match between item 3 and CRU as a concept, the other evidence sources rendered the scores and interpretations from this item as less valid which affects the overall validity of the CRU scale. We trust the formal content validity assessment finding that the item is a good match with CRU. However, we believe, as seen in the response processes evidence, that the healthcare aides in our sample had difficulty understanding the item, thus rendering their responses to it as less valid. Future work on this item is required and should entail in-depth response processes work with healthcare aides to ensure clarity in item wording without appreciable loss in meaning.

Relations with other variables evidence also added to the construct validity argument for the CRU scale. Statistically significant bivariate correlations (Table 5) between the CRU latent scale score and the five item's scores with instrumental, persuasive, and overall research utilization reinforce past empirical research [2,7], providing supporting validity evidence. The regression analysis (Table 6) also provided supporting validity evidence by showing that the CRU scale score was a predictor of overall research utilization, after controlling for other covariates [2,7].

### The Factor Model

While the items comprising the CRU scale were originally selected to cluster on one dimension (CRU) they were also intentionally selected to be non-redundant, allowing each item to focus on a slightly different feature of CRU. The intended 'clustering' of the items onto a factor renders the factor model the most appropriate model for assessing the internal structure of the CRU scale but the purposefully non-redundant nature of items meant that the scale would not function perfectly as a factor model. We employed three factor models: Model 1 with the five items loading onto a single factor, Model 2 with the five items loading onto a single factor with correlated errors between two sets of items (items 1 and 2, and items 3 and 4), and Model 3 with four items (item 1 was removed) loading onto a single factor with correlated errors between one set of items (items 3 and 4). A fourth model with one of items 3 or 4 also removed (in addition to item 1) would have been the next logical alternative model. However, this model would be just identified (*df *= 0) and thus, not testable. Item parceling (i.e., combining items into small groups of items within scales or subscales) has been used by others to deal with issues around local dependence and lack of unidimensionality. This was not an option here given the small number of items in the CRU Scale; by parceling items 3 and 4 along with removal of item 1, the model would remain 'just identified' and not testable.

As an alternative to the strict factor models assessed in this study, a model appropriately acknowledging the non-redundancy of the CRU items could be used. This would require use of single-item latent concepts, but such a model does not provide the kind evidence required by the *Standards*. A better model may be to simultaneously assess both measurement and latent structures using structural equation modeling. However, at this stage we do not know enough about the causal world of conceptual research utilization by healthcare aides to construct this model. Further research is needed to identify predictors of and outcomes to CRU, following which a causal model of CRU can be developed and tested. A CFA model was therefore our next best choice at this stage of the development of CRU with which to assess the internal structure of the CRU Scale.

## Limitations

Although the psychometric assessment reported in this paper is promising, the findings presented should be considered in light of the study's limitations. First, the study was conducted in one country with one group of healthcare providers from a single context - healthcare aides in nursing homes. Assessment of a new instrument is a multi-step process that requires multiple revisions and reassessment across a range of settings and provider groups. Second, our reliability assessment was limited to tests of internal consistency. Future applications of the CRU scale should examine scale stability (test-retest reliability) in addition to the scale's internal consistency. Third, the internal structure analyses revealed information about how each of the five items in the CRU scale relate to the latent concept of CRU. These findings suggest that research (using classical test score and item response theory) investigating approaches to deriving an overall latent score for the CRU scale (e.g., sum, mean, weighting) is needed. Fourth, we conducted the expert panel content validity assessment and the pilot test concurrently. This prevented us from making revisions to the scale based on the expert panel assessment before pilot testing the scale. Fifth, the data used in sample 3 (pilot test) of this study has a naturally occurring multi-level nature (individuals - units - nursing homes) which could have a biasing effect on the analyses reported here; the ICC(1) values for CRU scale score however revealed CRU is largely an individual concept in this dataset supporting our choice of analyses and limiting any potential bias in this regard. Finally, because this was the first administration of the CRU scale, it has not yet been used in studies of research utilization interventions. Therefore, it is not known whether the scale is sensitive to and able to detect changes in CRU over time. Despite these limitations, the CRU scale addresses an important gap in health services research - the ability to assess healthcare aides' conceptual use of research findings. To date, research utilization has been measured predominantly as an omnibus or general concept. Failure to measure CRU results in: (1) an underestimate of the extent to which healthcare providers use research in practice and, (2) a lack of understanding of the true research utilization process.

## Conclusions

The CRU scale assessed in this paper showed acceptable beginning psychometric properties with respect to responses from healthcare aides in nursing homes whose first language was English. The analyses of validity, reliability, and acceptability are promising. These findings, however, are not generalizable beyond healthcare aides in Canadian nursing homes that speak English as their first language. Based on our findings, we recommend only using the four-item version of the CRU scale (*i.e.*, without item 1: give new knowledge or information) to yield sound assessments of CRU by healthcare aides. Future research should first include exploration of item 1 as a possible distinguished aspect of CRU and revision to the wording of item 3 (help change your mind), followed by investigation of: (1) reasons for differences in CRU scale scores by first language spoken, (2) latent scale scoring, (3) variables that predict and are outcomes to CRU (e.g., resident and organizational outcomes), and (4) longitudinal work to determine whether the CRU Scale and its items are sensitive to changes in levels of CRU.

## Abbreviations

Abbreviations used in this manuscript include: (1) (CRU): Conceptual Research Utilization; (2) (TREC): Translating Research in Elder Care; (3) (EFA): Exploratory Factor Analysis; (4) (PCA): Principal Components Analysis; (5) (CFA): Confirmatory Factor Analysis; (6) (I-CVI): Item-Content Validity Index; (7) (S-CVI): Scale-Content Validity Index; (8) (RMSEA): Root Mean Square Error of Approximation; (9) (SRMR): Standardized Root Mean Square Residual; (10) (CFI): Comparative Fit Index; (10) (ICC): Intraclass Correlation.

## Competing interests

The authors declare that they have no competing interests.

## Authors' contributions

All authors participated in designing the study. JES and CAE developed the CRU scale. JES performed the statistical analysis and drafted the manuscript. CAE, CNC, and MG provided valuable advice throughout the study. All authors provided critical commentary on the manuscript and approved the final version.

## Pre-publication history

The pre-publication history for this paper can be accessed here:

http://www.biomedcentral.com/1472-6963/11/107/prepub

## Supplementary Material

Additional file 1 **The CRU Scale**. The CRU scale as presented to the expert panel and used in the pilot test Click here for file

Additional file 2 **CRU Scores by First Language**. A summary of scores on the CRU items and scale score according to whether or not English was the healthcare aides first language Click here for file

Additional file 3 **CFA Model 1 Additional Diagnostics**. A summary of standardized residuals and modification indices for CFA Model 1 Click here for file
