# Supplementary material for: Validation of the conceptual research utilization scale: an application of the standards for educational and psychological testing in healthcare
Source: BMC Health Serv Res. 2011 May 19;11:107. doi: 10.1186/1472-6963-11-107 (PMC3117685; doi:10.1186/1472-6963-11-107)
Supplement: Additional file 1 — The CRU Scale. The CRU scale as presented to the expert panel and used in the pilot test [file 1472-6963-11-107-S1.PDF]

Additional File 1:The CRU Scale

On your LAST typical work day on Unit ________, how often did best practice knowledge about things like pain management, preventing falls, and managing difficult behaviors do any of the following?

	Never	Rarely	Occasionally	Frequently	Almost Always
1. Give you new knowledge or information about how to care for residents.	❑ 1	❑ 2	❑ 3	❑ 4	❑ 5
2. Raise your awareness about new ways to care for residents.	❑ 1	❑ 2	❑ 3	❑ 4	❑ 5
3. Help to change your mind about how to care for residents.	❑ 1	❑ 2	❑ 3	❑ 4	❑ 5
4. Give you new ideas about how to care for residents.	❑ 1	❑ 2	❑ 3	❑ 4	❑ 5
5. Help you make sense of things you have been doing to care for residents.	❑ 1	❑ 2	❑ 3	❑ 4	❑ 5

This scale cannot be reproduced or used without written permission of the first two authors of this paper.
