# Supplementary material for: Validation of the conceptual research utilization scale: an application of the standards for educational and psychological testing in healthcare
Source: BMC Health Serv Res. 2011 May 19;11:107. doi: 10.1186/1472-6963-11-107 (PMC3117685; doi:10.1186/1472-6963-11-107)
Supplement: Additional file 2 — CRU Scores by First Language. A summary of scores on the CRU items and scale score according to whether or not English was the healthcare aides first language [file 1472-6963-11-107-S2.PDF]

Additional File 2. CRU Scores by First Language

Item	EFL[1]	Mean (STD Dev)	Degrees of Freedom	Significance (t-test)
**CRU Scale Score**	Yes<br>No	3.60 (0.891)<br>4.11 (0.692)	1346	< 0.001
**Item #1:** Give new knowledge or information	Yes<br>No	3.66 (1.058)<br>4.20 (0.892)	1346	< 0.001
**Item #2:** Raise awareness	Yes<br>No	3.69 (1.023)<br>4.25 (0.862)	1346	< 0.001
**Item #3:** Help change your mind	Yes<br>No	3.37 (1.142)<br>3.65 (1.266)	1346	< 0.001
**Item #4:** Give new ideas	Yes<br>No	3.57 (1.047)<br>4.13 (0.926)	1346	< 0.001
**Item #5:** Help make sense of things	Yes<br>No	3.76 (1.023)<br>4.34 (0.799)	1346	< 0.001

[1]EFL= English as first language
sample size: listwise deletion used. Yes (English as first language, n=697), No (English not first language, n=651)
