# Supplementary material for: Validation of the conceptual research utilization scale: an application of the standards for educational and psychological testing in healthcare
Source: BMC Health Serv Res. 2011 May 19;11:107. doi: 10.1186/1472-6963-11-107 (PMC3117685; doi:10.1186/1472-6963-11-107)
Supplement: Additional file 3 — CFA Model 1 Additional Diagnostics. A summary of standardized residuals and modification indices for CFA Model 1 [file 1472-6963-11-107-S3.PDF]

Additional File 3: CFA Model 1 Additional Diagnostics

Standardized Residuals

	**Item #1:** Give new knowledge or information	**Item #2:** Raise awareness	**Item #3:** Help change your mind	**Item #4:** Give new ideas	**Item #5:** Help make sense of things
**Item #1:** Give new knowledge or information	--	--	--	--	--
**Item #2:** Raise awareness	7.991	--	--	--	--
**Item #3:** Help change your mind	4.008	1.605	--	--	--
**Item #4:** Give new ideas	3.563	4.587	5.618	--	--
**Item #5:** Help make sense of things	1.498	1.779	0.354	3.492	--

Modification Indices for THETA-DELTA (measurement errors)

	**Item #1:** Give new knowledge or information	**Item #2:** Raise awareness	**Item #3:** Help change your mind	**Item #4:** Give new ideas	**Item #5:** Help make sense of things
**Item #1:** Give new knowledge or information	--	--	--	--	--
**Item #2:** Raise awareness	63.859	--	--	--	--
**Item #3:** Help change your mind	16.064	2.575	--	--	--
**Item #4:** Give new ideas	12.697	21.039	31.566	--	--
**Item #5:** Help make sense of things	2.243	3.165	0.125	12.196	--
